# Pattern of occurrence and treatment of impacted teeth at the Muhimbili National Hospital, Dar es Salaam, Tanzania

**DOI:** 10.1186/1472-6831-13-37

**Published:** 2013-08-06

**Authors:** Farizana Msagati, Elison NM Simon, Sira Owibingire

**Affiliations:** 1 Oral and Maxillofacial Unit, Muhimbili National Hospital, Dar es Salaam, Tanzania

**Keywords:** Impacted teeth, Pattern of occurrence, Muhimbili, Tanzania

## Abstract

**Background:**

Impacted teeth predispose to periodontal disease and dental caries of adjacent teeth resulting in pain, discomfort and loss of function. This study analyzed the pattern of occurrence of impacted teeth, associated symptoms, treatment and complications of treatment in patients who presented at the Muhimbili National Hospital, Tanzania.

**Method:**

This was a crossectional descriptive study which utilized notes and x rays of patients who were treated for impacted teeth at the Oral and Maxillofacial firm in Muhimbili National Hospital over five years, from January 2005 to August 2010. These records were retrieved and examined for the major complaint of the patient at presentation to hospital, demography, impacted tooth, type of impaction (for third molars), treatment offered and complications after treatment. Similar information was collected from all patients with impacted teeth attended in the same centre from 1^st^ September 2010 to 31^st^ August 2011.

**Results:**

A total of 896 patients (496 males and 400 females) treated for complaints related to impacted teeth were recorded. The male to female ratio was 1.2:1, age range of 16 to 85 years and a mean age of 28.9 years (SD = 9.5).

Slightly more than 84% of the patients presented with mandibular third molar impactions. Most (44.7%) of these patients had an impacted lower right third molar followed by those presenting with a lower left third molar impaction (39.7%). In 1.3% of the patients all the four third molars were impacted. Sixty nine (7.7%) patients had impacted upper 3^rd^ molars while 2% had impacted upper canines. Of the mandibular 3^rd^ molar impactions 738 (76%) were mesio-angular type, 87 (8.9%) horizontal type and 69 (7.1%) disto-angular.

Patients presented with a variety of complaints. About 85% of the patients presented to hospital due to varying degrees of pain. In 4.9% the detection of the impacted tooth/teeth was coincidental after presenting to hospital for other reasons not related to the impaction.

Majority of the patients with impacted mandibular third molars had carious lesions on the impacted teeth, neighbouring tooth or both. Four hundred and five (45.2%) patients had a carious lesion on one of the impacted teeth while 201(22.4%) patients had a carious lesion on the adjacent second molar. In 122 (13.6%) patients both the impacted third molar and the adjacent second molar were carious. In twelve patients who presented with a main complaint of fracture of the angle of the mandible there was an associated impacted 3^rd^ molar. Eight hundred and fifteen (91%) patients with impacted teeth were treated by surgical removal. Among these only 15 (1.8%) had complications that ranged from excessive swellings, trismus and severe pain post operatively. One patient was reported to have fracture of the angle of the mandible sustained during surgical removal of an impacted 48.

**Conclusions:**

The majority of patients with impacted teeth were young with an almost equal sex distribution. The most commonly impacted teeth were mandibular third molars followed by the maxillary third molars. Patients with impacted teeth reported for health care predominantly because of pain due to dental caries or infection.

There is a need of creating appropriate programmes that would further raise peoples’ awareness to regular dental checkups so that appropriate measures are taken before complications arise.

## Background

Impacted teeth fail to erupt fully into the oral cavity within the expected time due to impact with the jaw bone, adjacent tooth or even the gums [1-3]. Lack of an adequate dental arch length and space in which to erupt into is the main reason for this failure. Studies have shown the mandibular last molar to be the most commonly impacted tooth followed by the maxillary third molars, the maxillary canines and the mandibular premolars [3-7]. Impaction of the incisors is relatively rare compared to the other teeth and when present the cause is often a retained decidous tooth or the presence of another abnormality like an odontoma [8-10]. Multiple impactions are in most instances seen in association with some syndromes such as cleidocranial dysostosis, Gardner’s syndrome, Gorlin-Sedano syndrome and Yunis-Varon syndrome [11-15]. A case of impactions of primary teeth which is generally rare was reported, however, this was associated with tooth agenesis in a monozygous twin [16]. Impacted teeth are usually painless but when infections of surrounding tissues occur, severe pain result. Pressure on the inferior alveolar nerve in very deeply positioned lower third molar impactions may be another reason for pain [1,17]. Presence of impacted teeth predisposes the erupted adjacent teeth to periodontal disease and caries formation [18,19]. In Turkey, Mollaoglu (2002) in a study of volunteers found out that the mesiodistal angulation of the third molars was significantly greater while the retromolar space of the third molar was significantly smaller in the impacted group [20]. Chu et al. (2003) in a study of a Hong Kong Chinese population showed that eight percent of teeth adjacent to impacted mandibular third molars had periodontal loss of more than 5 mm and caries was found in the same surfaces in 7% of the adjacent second molars [4]. Symptoms associated with impacted mandibular third molars were reported in 30% of patients in Nigeria [17]. Oginini (*2002*) reported infections in patients with impactions ranging from pulpitis, pericoronitis and periodontitis [21]. Some studies reported that 96.1% of impacted mandibular third molars were removed under local anaesthesia while 2.9% were done under general anaesthesia and were often accompanied by some complications [17,22]. The only retrievable study done in Tanzania was epidemiological [23].

The aim of this study therefore was to analyze the pattern of occurrence of impacted teeth, associated symptoms, treatment and complications of treatment in patients presenting at the oral and maxillofacial unit of the Muhimbili National Hospital.

## Methods

This was a crossectional descriptive study which utilized notes and x rays of patients who were treated for impacted teeth at the Oral and Maxillofacial firm in Muhimbili National Hospital over five years, from January 2005 to August 2010. These records were retrieved and examined for the major complaint of the patient at presentation to hospital, demography, impacted tooth, type of impaction (for third molars), treatment offered and complications after treatment. Similar information was collected from all patients with impacted teeth attended in the same centre from 1^st^ September 2010 to 31^st^ August 2011.

Information collected was recorded in a special form. A tooth was considered to be impacted if it failed to erupt fully into the oral cavity. However, specifically for the 3^rd^ molars, x rays were further used to analyze the type of impaction while reference was made to the adjacent second molar [23-27]. Two lines, each drawn separately along the longitudinal axes of the adjacent second molar and the impacted 3^rd^ molar were used to determine whether the impaction was vertical, mesioangular, distoangular or horizontal [25,27]. Also, the relationship of the upper part of the crown of the impacted tooth to the occlusal surface of the adjacent fully erupted second molar was used to determine the levels of the impacted teeth. In patients who the second molar for any reason happened to be missing this classification was not used. The x rays were interpreted by a single person (ES- second author) who has vast experience in oral radiology. Intra examiner variability was done by randomly repeating 10% of the radiographs to ascertain consistency throughout the study with a reproducibility of 89%.

Treatment in all of the cases was carried out after a thorough analysis of the type of impaction, condition of the crown e.g. whether it was grossly carious, amount of overlying bone, proximity with the adjacent tooth and presence of infection. Horizontally impacted teeth required substantial bone removal and sectioning of the tooth. Except for patients with established infection, no medications were prescribed to patients prior to the anticipated surgery. However, post extraction analgesics and broad spectrum antibiotics were prescribed to all patients. Complications that arose during and after treatment were recorded.

Data was entered into computer and analyzed using SPSS programme (version 15). Frequency distribution was generated to describe the demographic characteristics of the study population, the different impacted teeth and types of mandibular 3^rd^ molar impactions and complications after treatment.

### Ethical issues

Ethical clearance for the study was issued by the research and publications committee of the Muhimbili University of Health and Allied Sciences through the Department of Preventive and Community Dentistry School of Dentistry. Patients were informed on the purpose of the research, assured of strict confidentiality and requested for their consent. Their consent to participate or not would not affect their management.

## Results

A total of 896 patients (496 males and 400 females) who reported to hospital and were found to have impacted teeth were recorded and included in the study (Table 1). The male to female ratio was 1.2:1, age range of 16 to 85 years and a mean age of 28.9 years (SD = 9.5).

**Table 1:** Distribution of the patients by age and sex

**Age group (yrs)**	**Male**	**Female**	**Total**
15 – 19	6	13	19
20 - 24	102	104	206
25 - 29	149	162	311
30 – 34	113	76	189
35 – 39	72	12	84
40 – 44	32	16	48
45 – 49	16	12	28
50 - 54	2	1	3
55 - 59	4	0	4
≥ 60	0	4	4
**Total**	**496 (55.4%)**	**400 (44.6%)**	**896**

Slightly more than 84% of the patients presented with mandibular third molar impactions. Most (44.7%) of these patients had an impacted lower right third molar followed by those presenting with a lower left third molar impaction (39.7%). In 1.3% of the patients all the four third molars were impacted. About 7.7% of the patients had an impacted upper 3^rd^ molar while 2% had an impacted upper canine. Of the mandibular 3^rd^ molar impactions 738 (76%) were mesio-angular type, 87 (8.9%) horizontal type and 69 (7.1%) disto-angular.

Patients presented with a variety of complaints (Table 2). About 85 per cent of the patients presented to hospital due to varying degrees of pain. In 4.9% the detection of the impacted tooth/teeth was coincidental after presenting to hospital for other reasons not related to the impaction.

**Table 2:** Patient’s different main presenting complaints

**Ser. No.**	**Presenting complaints**	**N**	**%**
1.	Mild pain	23	2.9
2.	Moderate pain	69	8.7
3.	Severe pain	587	73.8
4.	Pain and paraesthesia	15	1.9
5.	Pain and swelling	46	5.8
6.	Aesthetics	8	1.0
7.	Other	47	5.9
	**Total**	**896**	**100.0**

Majority of the patients with impacted mandibular third molars had carious lesions on the impacted teeth, neighbouring tooth or both. Four hundred and five (45.2%) patients had a carious lesion on one of the impacted teeth while 201(22.4%) patients had a carious lesion on the adjacent second molar. In 122 (13.6%) patients both the impacted third molar and the adjacent second molar were carious. In twelve patients who presented to hospital because of fracture of the mandible there was an associated impacted 3^rd^ molar. Eight hundred and fifteen (91%) patients with impacted teeth were treated by surgical removal. Among these only 15 (1.8%) had complications that ranged from excessive swellings and trismus and severe pain post operatively (Table 3). One among these patients was reported to have suffered fracture of the angle of the mandible sustained during surgical removal of an impacted 48.

**Table 3:** Complications that resulted from treatment of impacted teeth

**Ser No.**	**Complication**	**N**
1.	Severe pain	6
2.	Post operative swelling	4
3.	Trismus	2
4.	Swelling and trismus	2
5.	Fracture during the operation	1
**Total**		**15**

## Discussion

This was a hospital based study whereby information on patients who presented with complaints related to impacted teeth or those presenting for other complaints but coincidentally were found to have impacted teeth was collected. In some situations the impacted teeth, although presenting symptoms, could not be clearly visualized in the oral cavity making radiological examination the only means of reaching a substantive diagnosis and planning of appropriate management.

There were slightly more males than females who reported with impacted teeth, however, the age profile was almost similar for both (Table 1). The mean age of patients with impactions as was found in this study was slightly higher compared to reports from Europe and America [3,20]. This might be explained by the fact that in those countries impactions are discovered during routine dental examinations which start at younger ages [28-30]. On the other hand in resource poor countries and for Tanzania in particular, majority of the patients report for health care only after experiencing symptoms that lead to a certain degree of incapacitation which varies from mild to severe pain, swellings, trismus or fever.

**Figure 1:**
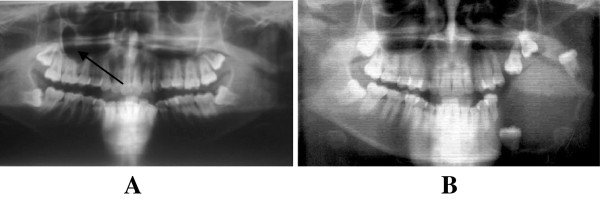
**Incidental findings in patients presenting with impacted teeth. A**. An OPG of a young patient who presented with a painless swelling on right side of the upper jaw (arrow) and was incidentally found to have all last molars impacted. **B**. A big pathological lesion possibly predisposed by presence of impacted teeth.

**Figure 2:**
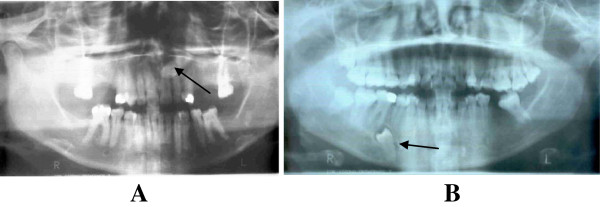
**Symptomless impacted teeth. A**. The buried upper left canine (arrow) was coincidentally found in this patient who presented with TMJ problems. **B**. Buried supernumerary lower right premolar (arrow) that had no symptoms. The same patient had impacted upper and lower right third molars.

**Figure 3:**
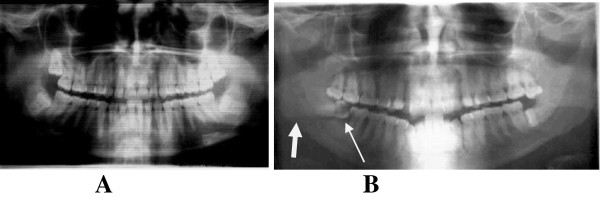
**Multiple impacted teeth. A**. An OPG of a 27 years old lady with all 3^rd^ molars impacted. She had no complaints. **B**. Bilateral mandibular 3^rd^ molar impactions. Both 47 and 48 are carious (narrow arrow) and the patient sustained fracture in the region of the lower right 3^rd^ molar (thick arrow).

In this study, the most (89.3%) commonly impacted teeth were mandibular 3^rd^ molars (Table 4). These findings are in concurrence with the findings from other studies [6,17,21,31]. The reasons for this occurrence have been explained by others [32,33]. There were few patients who were found to have multiple impactions (Figure 1A). Often these were coincidental findings when patients presented with either complaints from only one of them or a completely different complaint unrelated to the impactions (Figure 2). Impacted canines and premolars were a rarity (<15%) most possibly because when impacted these teeth often interfere with aesthetics therefore were removed at younger ages. Furthermore, patients with teeth that did not erupt into the oral cavity generally did not present any complaints but rather the findings were incidental. Conversely, impacted molars that partially appeared in the oral cavity were found to be responsible for a number of problems including severe pain from pulpitis due to caries or pericoronitis (Figures 1B and 3B).

**Table 4:** Distribution of patients according to the different types of impacted teeth

**Ser. No.**	**Impacted tooth/teeth**	**Frequency**	
		**n**	**%**
1.	Lower right third molar	402	44.7
2.	Lower left third molar	356	39.7
3.	Both lower left and lower right third molars	62	6.9
4.	Upper right third molar	21	2.4
5.	Upper left third molar	16	1.7
6.	Both upper right and upper left canines	11	1.2
7.	All four 3^rd^ molars	13	1.4
8.	Upper right canine	7	0.8
9.	Upper left canine	7	0.8
10.	Lower right first premolar	2	0.2
11.	Both lower right and left first premolars	2	0.2
**Total**		**899**	**100**

Most (76%) of the third molar impactions were mesio-angular, above or at the level of the cervical margin of the adjacent second molar. This poised several disadvantages; once a portion or the whole crown of the impacted tooth was exposed to the oral environment the interplay between food substrates and oral bacteria could easily lead to the development of caries. This is further aggravated by the fact that adequate cleanliness of the whole or part of the surface of the impacted tooth and the distal surface of the neighbouring tooth becomes almost impossible. This most possibly explains why in this study 46.6% of the patients were found to have caries on the impacted tooth and 36% had caries on the adjacent tooth (Figure 1).

In this study over 80% of the patients reported varying degrees of pain and incapacitation, with 73% experiencing severe pain (Table 2). Only 5% of the patients did not present any complaints at all. Pain mostly resulted from pulpitis due to deep carious lesions on the impacted tooth, the adjacent one or on both (Figure 3B). Delay in seeking appropriate medical or dental health care was the main reason for the severe pain that patients presented with. Delay in reporting for oral health care in Tanzanian communities has been attributed to ignorance, social-cultural factors and lack of adequate oral health care services in the country [34].

The fact that only 15 (1.8%) suffered complications after surgical treatment reflects a good success level in the methods of treatment applied in this centre (Table 3). Weakening of the crown due to gross caries, amount of overlying bone, proximity to the adjacent tooth and presence of infection were important factors taken into consideration during treatment planning. The position and depth of the impacted tooth, particularly the mandibular third molar, compromises the strength of this bone predisposing it to fractures at the angle (Figure 3A). In such situations therefore, surgical bone removal through drilling with a bur, tooth resection and elevation were done with great care.

Contrary to the practice elsewhere [2,35], and unless otherwise dictated by an underlying systemic condition, patients were not routinely given medication prior to the anticipated surgery. Swellings a day or two after surgery were accompanied by differing levels of trismus. This is similar to reports from other African and European studies [3,19,21,36]. Postoperative pain of different degrees was also one of the complaints of patients postoperatively. Similar to other studies in some cases this was because of either an infected or dry socket [21,36]. The pain was managed by reassurance, the use of common analgesics and obtundents. The patient who was reported to have suffered fracture at the angle of the mandible during treatment had a deeply lying horizontally impacted 3^rd^ molar. This emphasizes the need for an adequate sectioning of the tooth to avoid the necessity of the use of excessive force during removal [37].

## Conclusion

The majority of patients with impacted teeth were rather young with an almost equal sex distribution. The most commonly encountered impacted tooth was mandibular third molar followed by the maxillary third molars. Patients with impacted teeth reported for health care predominantly because of pain due to dental caries or infection and only in very few cases were the patients aware of the existence of the impactions.

Appropriate programmes that will further raise peoples’ awareness on the importance of regular dental checkups which could lead to an early detection of impacted teeth and institution of appropriate measures before complications arise are necessary.

## Competing interests

The authors declare that they have no competing interests whatsoever.

## Authors’ contributions

FM was the principal investigator and took part in the design of the study and data collection, ENMS assisted with the designing of the study, treatment of the patients and collection of data and SO treated some of the patients, took part in data collection and preparation of the manuscript. All authors read and approved the final manuscript.

## Authors’ information

FM was a researcher working for elective period study, ENMS and SO are specialist oral and maxillofacial surgeons and lecturers in the Department of Oral and Maxillofacial Surgery of the Muhimbili University of Health and Allied Sciences.

## Pre-publication history

The pre-publication history for this paper can be accessed here:

http://www.biomedcentral.com/1472-6831/13/37/prepub
